# Design specification management with automated decision-making for reliable optimization of miniaturized microwave components

**DOI:** 10.1038/s41598-022-04810-1

**Published:** 2022-01-17

**Authors:** Slawomir Koziel, Anna Pietrenko-Dabrowska, Piotr Plotka

**Affiliations:** 1 Engineering Optimization and Modeling Center, Reykjavik University, 102 Reykjavik, Iceland; 2 Faculty of Electronics, Telecommunications and Informatics, Gdansk University of Technology, 80-233, Gdansk, Poland

**Keywords:** Electrical and electronic engineering, Computational science

## Abstract

The employment of numerical optimization techniques for parameter tuning of microwave components has nowadays become a commonplace. In pursuit of reliability, it is most often carried out at the level of full-wave electromagnetic (EM) simulation models, incurring considerable computational expenses. In the case of miniaturized microstrip circuits, densely arranged layouts with strong cross-coupling effects make EM-driven tuning imperative to achieve the optimum performance. The process is even more challenging due to a typically large number of geometry parameters, and the lack of reasonable initial designs. The latter often encourages the use of global search procedures, which may be prohibitively expensive. In this paper, a novel automated framework for reliable optimization of miniaturized microwave components is proposed. Our methodology is based on design specification management, where the performance requirements imposed on the system are temporarily relaxed if the current design is unlikely to be improved (e.g., due to being away from the target operating frequency). The specifications are re-adjusted at each iteration of the algorithm, and eventually converge to their original values. Using two examples of compact microstrip couplers and a power divider, the presented technique is demonstrated to significantly improve the efficacy of local search routines under challenging design scenarios.

## Introduction

Over the recent years, numerical optimization has been playing an increasing role in the design of high-frequency systems, including microwave components and devices^1–8^. This shift from traditional methods largely based on interactive parameter sweeping, has been motivated by a practical necessity. Tight performance requirements imposed on modern microwave systems, partially dictated by the demands pertinent to emerging application areas, e.g., wireless sensing^9^, Internet of Things (IoT)^10^, microwave imaging^11^, 5G^12^, autonomous vehicles^13^, wearable devices^14^, can only be met if meticulous development of the circuit architecture (e.g., geometry in the case of microstrip components) is supplemented by careful tuning of its parameters. Simultaneous adjustment of multiple variables while accounting for several performance specifications and constraints requires rigorous numerical procedures. To ensure reliability, especially at the final stages of the design process, optimization needs to be conducted at the level of full-wave electromagnetic (EM) simulation models. EM-driven design is of paramount importance especially for systems where simpler performance evaluation methods (e.g., equivalent network models) lack the accuracy. Representative examples include miniaturized microstrip components^15–18^, often implemented by folding conventional transmission lines (TLs)^19,20^, replacing TLs by compact microstrip resonant cells (CMRCs)^21,22^, or incorporation of the various topological alterations (e.g., defected ground structures^23^, substrate integrated waveguides^24^, stubs^25^, slots^26^, etc.). All of these result in strong cross-coupling effects, and the increased number of geometry parameters. This adds another layer of complexity to the design closure task, already challenging due to high CPU costs entailed by massive EM evaluations of the system at hand required by conventional optimization procedures.

Improving computational efficiency of simulation-based design procedures has been targeted by numerous research endeavours. These efforts focused on the development of strictly algorithmic approaches, both intrusive (e.g., gradient-based procedures accelerated by means of adjoint sensitivities^27,28^), and non-intrusive (e.g., trust-region methods with sparse sensitivity updates^29,30^, as well as surrogate-based frameworks involving data-driven^7^, and physics-based metamodels^5^. Although approximation surrogates (kriging^31^, radial-basis functions^32^, support vector regression^33^, polynomial chaos expansion^34,35^, neural networks^36^, Gaussian process regression^37^, polynomial regression^38^) are by far more popular, their application is limited by the curse of dimensionality, which is particularly troublesome when handling nonlinear outputs of high-frequency structures. Physics-based surrogates exhibit certain tolerance with this respect but require careful selection (and, clearly, availability) of an underlying low-fidelity model. Some popular methods of this category include space mapping^39^, cognition-driven design^40^, or various response correction methods^41,42^. A related class of techniques are those based on variable- and multi-fidelity simulations, e.g., co-kriging^43^, multi-fidelity procedures^44^, as well as optimization frameworks involving supervised learning^45,46^.

Although computational speedup is important, securing robustness of the optimization process may be even more essential when solving practical EM-driven design tasks. This occurs, in particular, when reasonably good initial designs are not available, e.g., in the case of re-designing the structure for a significantly different operating frequencies/bandwidths, or when tuning the parameters of a compact structure involving abbreviated components such as CMRCs^21^, or folded TLs^19^. On the one hand, local optimization starting from a poor initial point is likely to fail. On the other hand, engaging global search routines usually turns computationally expensive, often prohibitive. As mentioned before, the various algorithmic solutions outlined in the previous paragraph^5,7,27–46^ are mainly developed to reduce the CPU cost of the EM-driven design procedures with little emphasis on the robustness. Improved reliability can be achieved, to a certain extent, using surrogate-assisted versions of global search routines (mostly population-based metaheuristics^47,48^). However, applicability of these methods is seriously hindered by the curse of dimensionality, as well as high nonlinearity of microwave circuit characteristics. In practice, only a few independent parameters can be efficiently handled^49,50^; beyond that, construction of an accurate metamodel covering sufficiently broad ranges of the system variables may become infeasible.

One of the practical issues of microwave design closure is that performance requirements, especially in terms of the target operating frequencies, are often away from those at available initial point (current design), which makes the optimization process fail when using local search techniques. In practice, addressing this problem boils down to altering the specifications and performing repetitive optimization runs while gradually moving the requirements towards the original targets. This approach, while commonly used, is laborious, requires designer interaction and experience-driven specification adjustments. In this paper, a novel technique for enhancing the reliability of local optimization routines through intelligent decision-making has been proposed. Our methodology involves a knowledge-based design specification management scheme, which allows for automated adjustment of the optimization goals based on the detected discrepancies between the actual operating frequencies/bandwidths of the component at hand, and the target ones. Initially, the objectives are shifted into the vicinity of the actual operating conditions of the system, then continuously re-adjusted during the optimization process, and finally converging to their original values. Several benefits of this approach can be identified. First, the optimization algorithm becomes less sensitive to the quality of the initial design. Second, dimension scaling (re-design) of microwave components becomes realizable by means of local methods within broad ranges of operating frequencies. Furthermore, global search procedures are no longer necessary in situations that normally require resorting to such techniques, which indirectly results in reducing the costs of the parameter tuning procedures. Finally, the standard algorithms (e.g., gradient-based routines) can be used in many situations previously fostering utilization of advanced algorithmic frameworks such as surrogate-assisted or sophisticated machine learning methods. The aforementioned advantages have been demonstrated using three microstrip structures, two compact branch-line couplers (a single- and a dual-band), and dual-band power divider, with satisfactory designs rendered through gradient-based optimization with the initial designs being away from the design targets. The presented methodology can be considered a simple yet powerful way of improving the efficacy and reliability of design closure procedures for miniaturized microwave components.

## Design specification management for reliable microwave optimization

The purpose of this section is to introduce the design specification adjustment scheme with automated decision-making, as a tool for improving the reliability of microwave design optimization procedures. The specification management concept is a generic one. However, to enable its demonstration, it is combined with a particular local optimization algorithm, here, the trust-region gradient-based search procedure. The remaining part of this section is organized as follows. In “Specification management concept”, we motivate and outline the design specification adjustment concept that is based on the convergence status of the optimization process. Specification adjustment prerequisites” discusses the main assumptions and prerequisites for the adjustment process, whereas the detailed procedure is formulated in “Specification adjustment procedure”. Optimization engine: Trust-region algorithm” briefly recalls the trust-region algorithm, whereas the complete optimization algorithm is summarized in “Optimization algorithm”.

### Specification management concept

We aim at alleviating the difficulties of simulation-based parameter tuning of miniaturized microwave components, especially in situations where reasonable initial designs are not readily available. The latter is particularly important when using local optimization algorithms. A representative scenario is re-design (or dimension scaling) of a given structure for operating frequencies that are significantly shifted from those at the currently available design. Under such circumstances, gradient-based and similar routines are likely to fail, whereas resorting to global procedures (typically, population-based metaheuristics^51^, efficient global optimizers^52^, or machine learning methods^53^) entails considerable computational expenses.

In order to explain the automated design specification adjustment concept presented in this work, we start by formulating the EM-driven parameter tuning task. Let us assume that the structure of interest is supposed to operate at the target frequencies *f*_*k*_, *k* = 1, …, *N*, where *N* is the number of operating bands. Given the vector of design variables (typically, geometry parameters) ***x***, the EM-simulated system outputs ***S***(***x***) (typically, *S*-parameters versus frequency), and the target operating frequency vector ***F*** = [*f*_1_ … *f*_*N*_]^*T*^, the design task is defined as a minimization problem

1 $$ {\mathbf{x}}^{*} = \arg \mathop {\min }\limits_{{\mathbf{x}}} U({\mathbf{S}}({\mathbf{x}}),{\mathbf{F}}) $$

where *U* is an objective function that quantifies the design quality. For example, let us assume that the circuit at hand is a microstrip coupler supposed to operate at the frequency *f*_0_. The circuit is to provide equal power split, while minimizing the matching and isolation, both at *f*_0_. Thus, the relevant system outputs would be *S*_11_, *S*_21_, *S*_31_, and *S*_41_, and the objective function can be defined as

2 $$ \begin{aligned} U({\mathbf{S}}({\mathbf{x}}),{\mathbf{F}}) & = U\left( {[S_{11} ({\mathbf{x}},f),S_{21} ({\mathbf{x}},f),S_{41} ({\mathbf{x}},f),S_{41} ({\mathbf{x}},f)],[f_{0} ]} \right) \\ & = \max \left\{ {|S_{11} ({\mathbf{x}},f_{0} )|,|S_{41} ({\mathbf{x}},f_{0} )|} \right\} + \beta \left[ {|S_{21} ({\mathbf{x}},f_{0} )| - |S_{31} ({\mathbf{x}},f_{0} )|} \right]^{2} \\ \end{aligned} $$

In this example, minimization of matching and isolation is our primary objective, whereas the power split condition is treated as a design constraint, and handled by adding a penalty term with the proportionality coefficient *β*.

**Figure 1:**
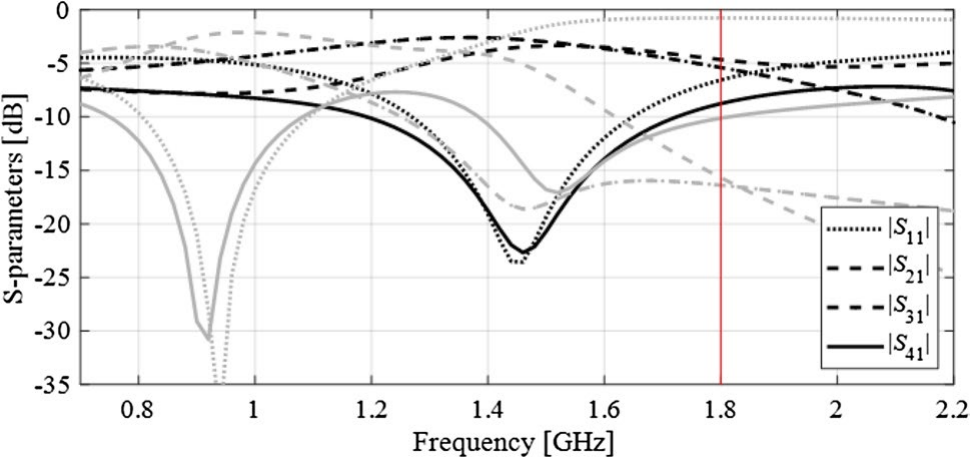
Scattering parameters of an example compact branch-line coupler (consider in “Example 1: Miniaturized branch-line coupler (BLC)”). The target operating frequency is 1.8 GHz, marked using a vertical line. This target is attainable by means of local search when starting from the design represented using the black lines; however, it is not attainable from the design marked using the gray lines. The latter is due to the fact that the operating bandwidth of the circuit is too far away from the target frequency.

Figure 1 illustrates a common situation where local optimization is prone to a failure due to the poor quality of the initial design. Among the two designs shown in the picture, the operating frequency of the first one (black lines) is sufficiently close to the target, whereas the second design (gray lines) is too far away in terms of the frequency misalignment to make the target attainable when using, e.g., a gradient-based search procedure.

The primary goal of this work is to develop algorithmic tools that permit reliable parameter tuning under challenging scenarios such as the one presented in Fig. 1 without defaulting to global optimization methods. Another objective is to avoid excessive computational costs, which would be entailed by straightforward approaches such as multiple optimization runs initiated from random starting points. Here, we propose a design specification adjustment strategy, where the target operating frequencies (or bandwidths) are altered by taking into account the actual operating frequencies of the structure at hand at the currently available design. The prerequisite for such alterations is to ensure that the optimum design with respect to the current status of the specifications is attainable at each stage of the optimization process. Another prerequisite is that the specifications eventually converge to their original levels towards the end of the procedure.

**Figure 2:**
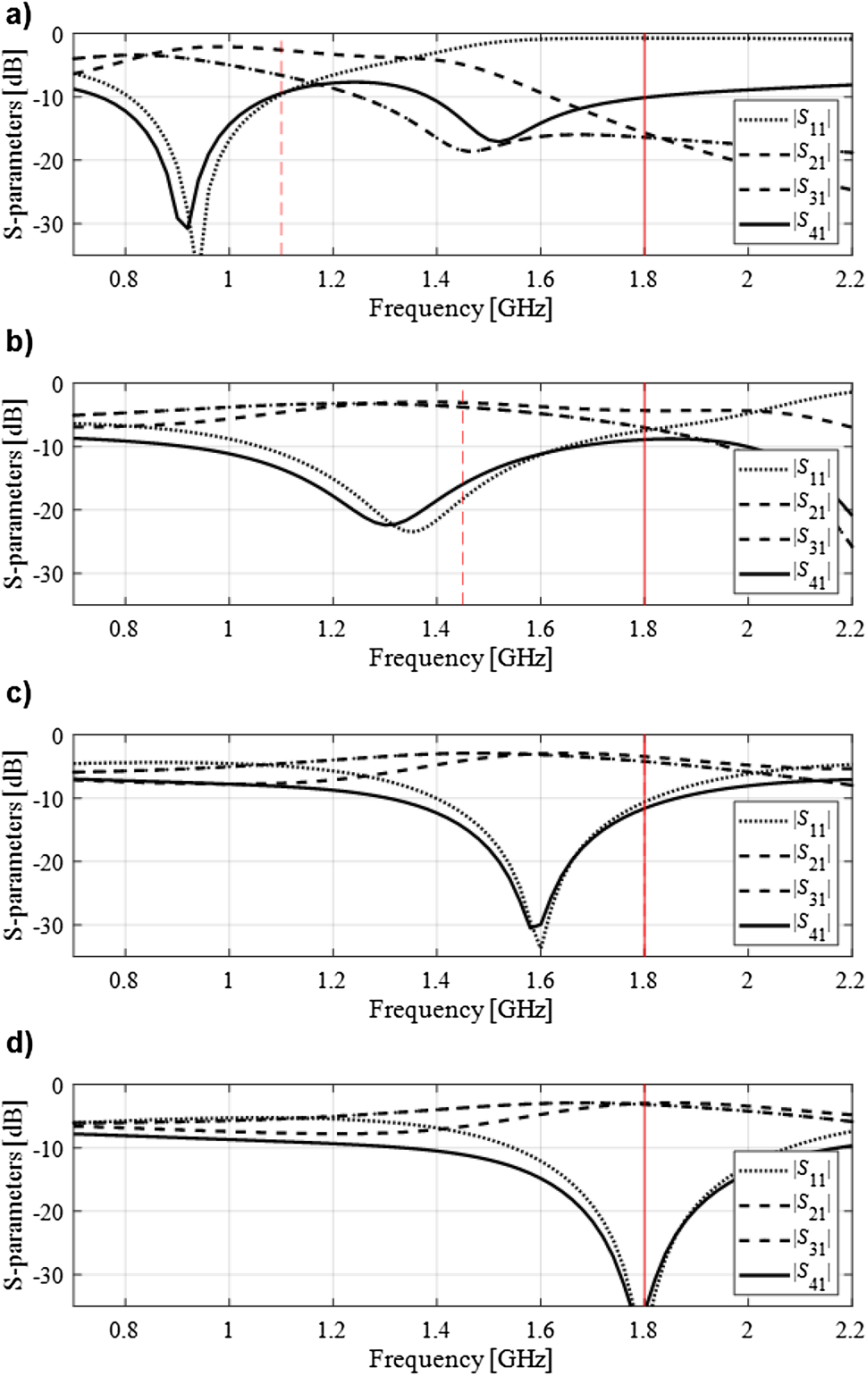
Automated design specification adjustment concept explained using a branch-line coupler. The initial design and the target operating frequency are the same as in Fig. 1 (initial design marked gray): (**a**) target frequency relocated towards the operating frequency at the initial design in order to ensure that the current specification (dashed line) are attainable from that design, (**b**) one of the iterations in the middle of the optimization run with the current design and current specifications, (**c**) final optimization stage; the target operating frequency converged to its original value, (**d**) final design optimized with respect to the original design requirement.

The aforementioned concepts have been illustrated in Fig. 2, and will be rigorously formulated in “Specification adjustment prerequisites” and “Specification adjustment procedure”. At the beginning of the optimization run, the target frequency is shifted to make the temporary goal attainable from a given initial design using a local algorithm. During the optimization process, the specifications are continuously adjusted based on the system outputs at the current design (cf. Fig. 2b,c). At the last stages, when the circuit operates sufficiently close to the original target frequency (or frequencies), the specifications are relocated accordingly so that the optimum design can be identified, again, using the local means. The adjustment of the specifications through automated decision-making process, and the design update (e.g., within the descent type of algorithm) are interleaved so that the entire procedure is concluded within a single algorithm run. It should be noted, that gradual alteration of the design specifications towards the ultimate targets throughout the optimization run is a common practice, when good initial designs are not available. This facilitates identification of the solutions meeting the assumed targets, which would otherwise be unattainable using the local search routines. Nevertheless, such an interactive process is heavily based on engineering insight, it is time consuming, and prone to failure. The approach presented in this work is fully automated, and requires no interaction from the designer. The knowledge necessary to make decisions concerning the scale of the adjustments, and the time of launching them, is acquired from the convergence indicators of the optimization process, design quality (as compared to the current targets), and the initial assessment of the frequency spread of the system outputs.

### Specification adjustment prerequisites

“Specification management concept” discussed the motivation for, the purpose, and potential benefits of involving intelligent decision-making in design specification adjustment. When it comes to its rigorous formulation, one needs to consider the following issues:

- The necessary amount of specification adjustment has to be quantified based on the available (current) design rendered during the optimization process;
- The (local) attainability of the adjusted specifications has to be determined when using the current design as the starting point;
- The above endeavours should be accomplished at low computational cost, preferably without involving extra EM simulations, apart from those already entailed by the operation of the core optimization algorithm itself.

The last factor is dictated by the practical utility of the procedure. Specific arrangements depend on the underlying optimization algorithm, which, in general, would be a type of a gradient-based routine. In this work, we use trust-region gradient search; consequently, the fundamental tool utilized to address the aforementioned issues will be the first-order Taylor expansion of the system frequency characteristics constructed using the sensitivity matrix, which is available at no extra cost: Jacobian has to be updated before each iteration of the trust-region algorithm as a part of its operation.

Let ***J***(***x***) denote the sensitivity matrix of all relevant system responses at the design ***x***. Without loss of generality, we can assume that the underlying optimization algorithm is an iterative procedure, which yields a series of approximations ***x***^(*i*)^, *i* = 0, 1, …, to the optimum design x^*^ of (1); here, ***x***^(0)^ is the initial design. Consider the first-order Taylor model *L*^(*i*)^(***x***) of ***S***(***x***) established at the current design ***x***^(*i*)^. We have

3 $$ L^{(i)} ({\mathbf{x}}) = {\mathbf{S}}\left( {{\mathbf{x}}^{(i)} } \right) + {\mathbf{J}}\left( {{\mathbf{x}}^{(i)} } \right) \cdot \left( {{\mathbf{x}} - {\mathbf{x}}^{(i)} } \right) $$

Furhter, let us consider a supplementary optimization problem

4 $$ {\mathbf{x}}^{tmp} = \arg \,\mathop {\min }\limits_{{\left\| {{\mathbf{x}} - {\mathbf{x}}^{(i)} } \right\| \le D}} U\left( {L^{(i)} \left( {\mathbf{x}} \right),{\varvec{F}}} \right) $$

In (4), *D* is the search radius in the vicinity of ***x***^(*i*)^, typically set to *D* = 1.

The knowledge-based adjustment of the design specifications will be determined using the following factors:

- The improvement factor *F*_*r*_ defined as
  5 $$ F_{r} = \left| {U\left( {L^{(i)} \left( {{\mathbf{x}}^{tmp} } \right),{\varvec{F}}} \right) - U\left( {L^{(i)} \left( {{\mathbf{x}}^{(i)} } \right),{\varvec{F}}} \right)} \right| $$
- The distance *D*_*c*_ between the actual operating frequencies of the system at hand at the desing ***x***^(*i*)^, denoted as ***F***_*c*_ = [*f*_*c*.1_ … *f*_*c*.*N*_]^*T*^, and the target frequencies ***F*** = [*f*_1_ … *f*_*N*_]^*T*^, defined as
  6 $$ D_{c} = \left\| {{\varvec{F}}_{c} - {\varvec{F}}} \right\| $$

The first factor, *F*_*r*_, allows us for assessing the potential for design improvement when starting from the current design ***x***^(*i*)^. The second factor, *D*_*c*_, is essentially a safeguard, introduced to ensure that the adjusted specifications are sufficiently close to the actual operating frequencies of the circuit under optimization at the current design. For both factors, the acceptance thresholds are defined: *F*_*r.*min_ and *D*_*c.*max_ (a practical procedure for setting them up will be discussed later). Using these, the design specification will be adjusted if:

- *F*_*r*_ < *F*_*r*.min_, i.e., the current design is unlikely to be improved to a sufficient extent, when starting from the current point ***x***^(*i*)^, or
- *D*_*c*_ > *D*_*c*.max_, i.e., the operating frequencies at the design ***x***^(*i*)^ are too far away from the current targets.

If either of these conditions is satisfied, the current specifications are too strict to be attainable from ***x***^(*i*)^, and should be relaxed. The acceptance threshold values are not critical; however, it is recommended that the system characteristics are taken into account, especially when deciding about the value of *D*_*c.*max_. In the following, we provide a simple yet practical procedure for establishing *F*_*r.*min_ and *D*_*c.*max_:

1. Set *D*_*c.*max_ to approximately half of the system bandwidth(s) at the initial design. This allows to allocate the target frequency (or frequencies) on the slopes of the frequency characteristics near the respective operating bandwidths if *D*_*c*_ < *D*_*c*.max_;
2. Relocate the target operating frequencies so that *D*_*c*_ = *D*_*c*.max_ (cf. (6));
3. Solve (4) with *D* = 1;
4. Set *F*_*r*.min_ = *F*_*r*_, with *F*_*r*_ calculated using the temporary design ***x***_*tmp*_ obtained in Step 3.

Establishing the threshold *F*_*r*.min_ as described above accounts for a typical improvement of the objective function assuming that design requirements are adjusted to satisfy the condition *D*_*c*_ < *D*_*c*.max_. As mentioned before, the latter is set to ensure that the operating frequencies of the system can be relocated to the current target frequencies using local search. When using these thresholds for adjusting the target operating frequencies within the actual optimization procedure, the above attainability property is therefore secured at each iteration of the algorithm.

### Specification adjustment procedure

We denote by ***F***_*current*_(*a*) = [*f*_*current.*1_(*a*) … *f*_*current.N*_(*a*)]^*T*^ the updated specifications (target frequencies) for the subsequent, or (*i* + 1)th, iteration of the optimization procedure. The vector ***F***_*current*_ is parameterized by a scalar *a*, 0 ≤ *a* ≤ 1. We have

7 $$ f_{current.k} (a) = (1 - a)f_{c.k} + af_{k} \quad {\text{for}}\quad k = 1, \ldots ,N $$

Recall that *f*_*c*.*k*_ are the entries of the vector ***F***_*c*_ = [*f*_*c*.1_ … *f*_*c*.*N*_]^*T*^ of the actual operating frequencies at the design ***x***^(*i*)^. The coefficient a is identified as the maximum number *a* ≤ 1 for which the condition *F*_*r*_ ≥ *F*_*r*.*min*_, and *D*_*c*_ ≤ *D*_*c*.*max*_, are satisfied at the design ***x***^*tmp*^ found as (cf. (4))

8 $$ {\mathbf{x}}^{tmp} = \arg \,\mathop {\min }\limits_{{\left\| {{\mathbf{x}} - {\mathbf{x}}^{\left( i \right)} } \right\| \le 1}} U\left( {L^{(i)} ({\mathbf{x}}),{\varvec{F}}_{current} (a)} \right) $$

The meaning of the last statement is that the coefficient *a* is, as a matter of fact, obtained through an auxiliary optimization procedure, in which it is adjusted (lowered) as much as necessary to eventually ensure that *F*_*r*_ ≥ *F*_*r*.min_ and *D*_*c*_ ≤ *D*_*c*.max_ for the vector ***x***^*tmp*^ generated by (8). At this point, the specifications have been relaxed to ensure that the target operating frequencies are sufficiently close to those at the design ***x***^(*i*)^ (in the sense of (6)), and the local improvement of the design (when starting from ***x***^(*i*)^) is at least equal to *F*_*r.*min_. As discussed before, satisfaction of these conditions make the adjusted specs attainable from ***x***^(*i*)^, and, consequently, throughout the entire optimization process.

Formally speaking, the updated frequency *f*_*current.k*_ is a convex combination of *f*_*c.k*_ and the original frequency *f*_*k*_. Reducing *a* leads to relaxing the specifications. When the current design approaches the optimum, the conditions will be satisfied for *a* = 1, i.e., the value to which the coefficient should eventually converge. The latter will take place if the original specifications are attainable. If this is not the case, the optimization algorithm will be terminated after approaching the target frequencies as closely as possible.

What was described so far in this section pertains to design specification adjustment in one algorithm iteration. The automated adjustment procedure is launched before each iteration, that is, at all points ***x***^(*i*)^, *i* = 0, 1, … This results in a continuous modification of the target frequencies based on the current relationships between the system response at ***x***^(*i*)^ and design targets. At the same time, it should be reiterated that the specification management is not associated with extra computational overhead (here, entailed by additional EM simulations of the system under design): the only required information is the sensitivity matrix, which is available anyhow as its evaluation is a part of the algorithm operation (as mentioned before, gradient-based routines are assumed in this work as the core optimization procedures).

### Optimization engine: trust-region algorithm

The design specification management strategy presented in this section can be incorporated into various iterative search procedures. Here, for the sake of demonstration, it is combined with the trust-region (TR) gradient search algorithm^54^. For the convenience of the reader, it is briefly outlined below. The problem at hand is the minimization task (1), and the algorithm produces a series of approximations ***x***^(*i*)^, *i* = 0, 1, …, to x^*^ (cf. (1)), by solving

9 $$ {\mathbf{x}}^{(i + 1)} = \arg \,\mathop {\min }\limits_{{\left\| {{\mathbf{x}} - {\mathbf{x}}^{(i)} } \right\| \le d^{(i)} }} U\left( {L^{(i)} ({\mathbf{x}}),{\varvec{F}}_{current} } \right) $$

where *L*^(*i*)^ is a linear model (3), ***F***_*current*_ represents the current vector of target operating frequencies, whereas trust region radius *d*^(*i*)^ is updated after each iteration of the algorithm using the gain ratio *r* = [*U*(***S***(***x***^(*i*+1)^), ***F***_*current*_) − *U*(***S***(***x***^(*i*)^), ***F***_*current*_)]/[*U*(*L*^(*i*)^(***x***^(*i*+1)^), ***F***_*current*_) − *U*(*L*^(*i*)^(***x***^(*i*)^), ***F***_*current*_)]; *r* > 0 indicates the design improvement, in which case ***x***^(*i*+1)^ is accepted. Furthermore, if *r* is large (e.g., *r* > 0.75), *d*^(*i*+1)^ is updated to 2*d*^(*i*)^; if *r* is too low (e.g., *r* < 0.25), *d*^(*i*+1)^ is reduced to *d*^(*i*)^/3; finally, if *r* < 0, the new design is rejected, and the iteration is repeated with a reduced TR. The example objective function has been discussed in “Specification management concept” (cf. (2)).

### Optimization algorithm

**Figure 3:**
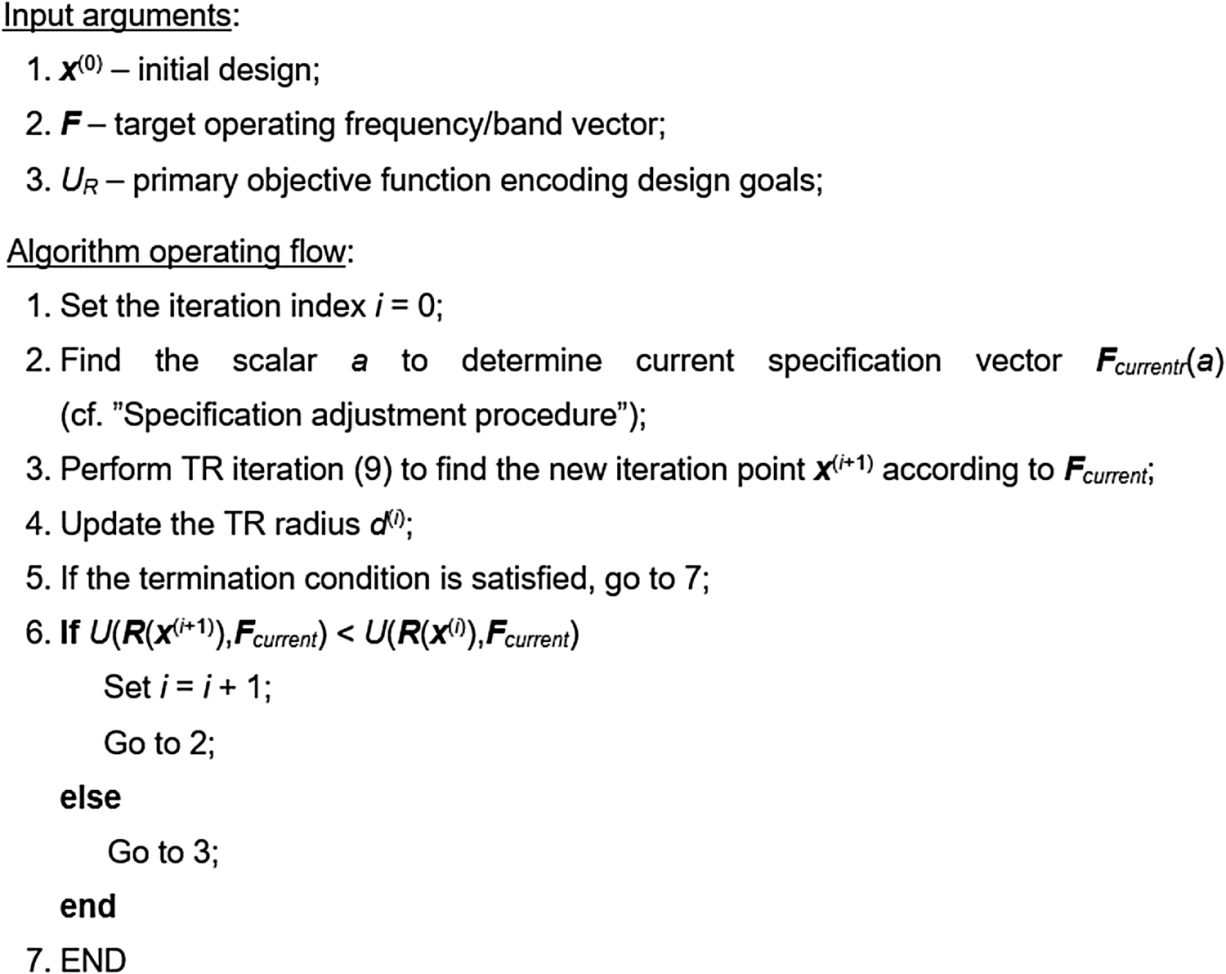
Pseudocode of the trust-region algorithm with design specification management scheme through intelligent decision-making of “Specification adjustment procedure”.

**Figure 4:**
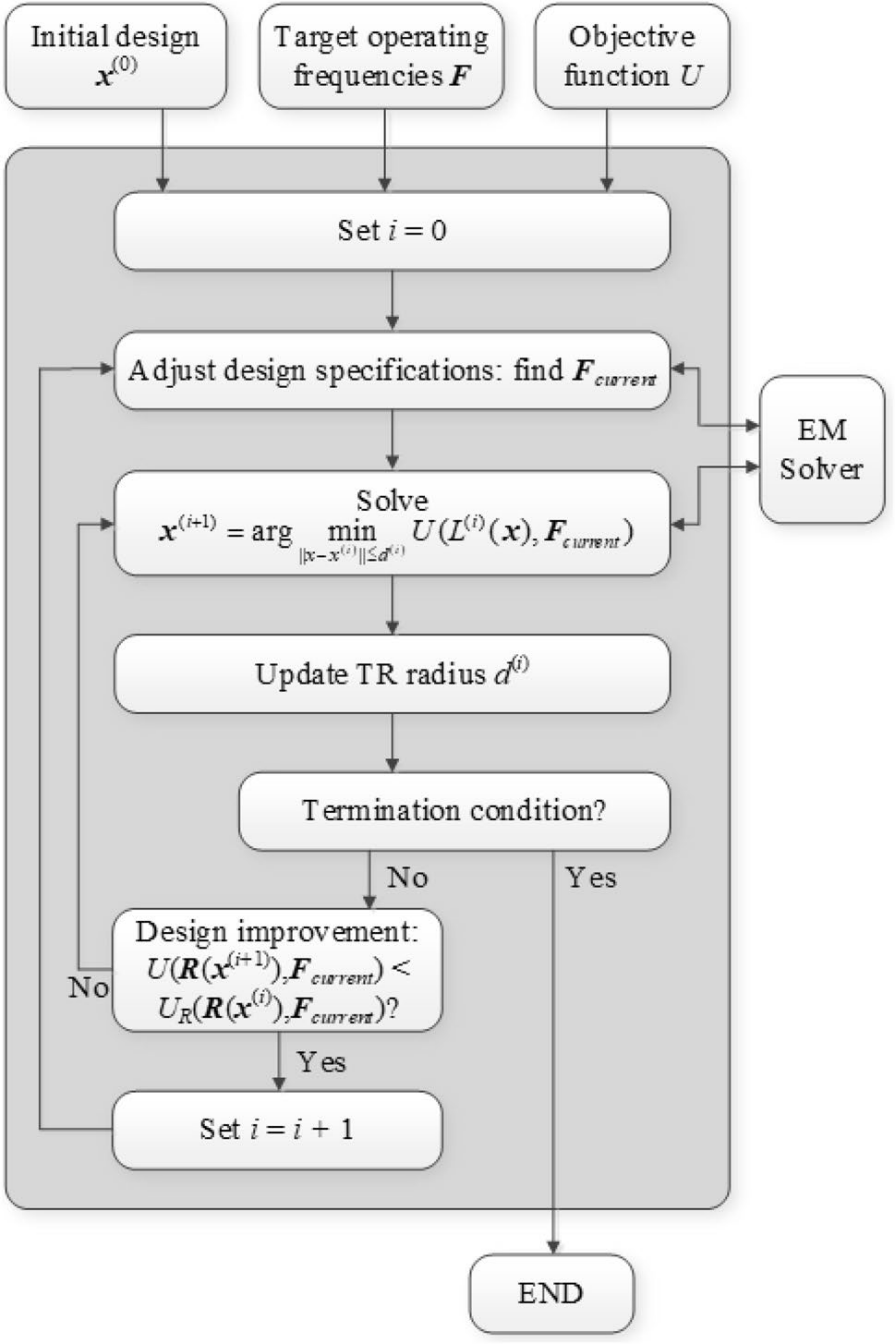
Flow diagram of the proposed optimization with design specification management through intelligent decision-making.

This section provides a description of the complete optimization procedure that combines local optimizer (here, the trust-region algorithm recalled in “Optimization engine: Trust-region algorithm”), and the design specification adjustment procedure of “Specification adjustment procedure”. The algorithm termination is based on the convergence in argument ||***x***^(*i*+1)^ − ***x***^(*i*)^||< ε, or reduction of the TR radius *d*^(*i*)^ < ε. The threshold ε is set to 10^−3^ in all numerical experiments presented in “Demonstration examples”. It should be noted that in the case of the lack of improvement of the objective function (cf. Step 6), the candidate design is rejected, and the iteration is repeated upon reducing the trust region radius. Interested readers can find more details concerning the trust-region algorithms in in the literature^54^. The pseudocode of the proposed optimization procedure, as well as the flow diagram have been shown in Figs. 3 and 4, respectively.

## Demonstration examples

In this section, the design specification management scheme introduced in “Design specification management for reliable microwave optimization” is demonstrated using three examples of miniaturized microstrip components, including two branch-line couplers (a single- and a dual-band ones), and a dual-band power divider. The numerical experiments are focused on emphasizing the benefits of the specification adjustment, as well as its importance in addressing the challenges related to the lack of quality initial designs.

### Example 1: miniaturized branch-line coupler (BLC)

**Figure 5:**
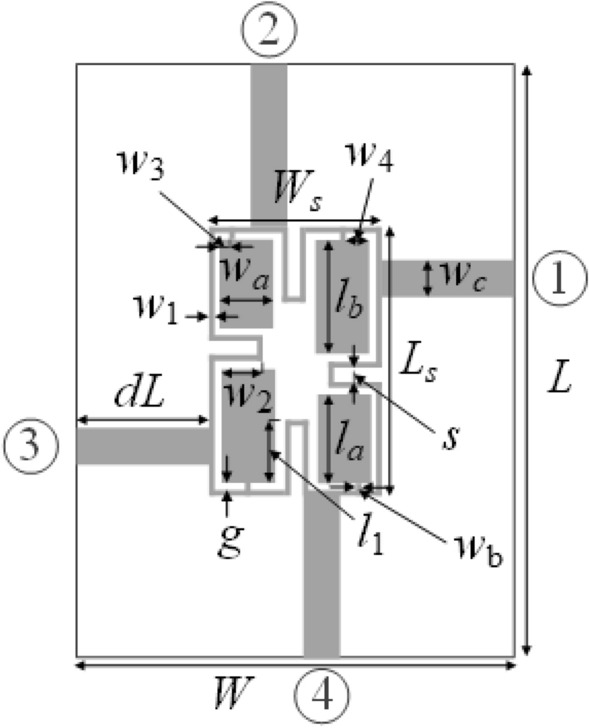
Miniaturized branch-line coupler (BLC)^55^. The circuit ports marked using numbered circles.

The first verification example is a miniaturized branch-line coupler (BLC)^55^. The circuit geometry is shown in Fig. 5. The structure is implemented on RO4003 substrate (*ε*_*r*_ = 3.5, *h* = 0.76 mm, tan*δ* = 0.0027). The design variables are ***x*** = [*g* *l*_1*r*_ *l*_*a*_ *l*_*b*_ *w*_1_ *w*_2*r*_ *w*_3*r*_ *w*_4*r*_ *w*_*a*_ *w*_*b*_]^*T*^. Other parameters are described by the following relations: *L* = 2*dL* + *L*_*s*_, *L*_*s*_ = 4*w*_1_ + 4*g* + s + *l*_*a*_ + *l*_*b*_, *W* = 2*dL* + *W*_*s*_, *W*_*s*_ = 4*w*_1_ + 4*g* + *s* + 2*w*_*a*_, *l*_1_ = *l*_*b*_*l*_1*r*_, w_2_ = *w*_*a*_*w*_2*r*_, *w*_3_ = *w*_3*r*_*w*_*a*_, and *w*_4_ = *w*_4*r*_*w*_*a*_. The computational model of the structure is implemented in CST Microwave Studio and simulated using the frequency domain solver (~ 60,000 mesh cells, simulation time about 5 min).

**Figure 6:**
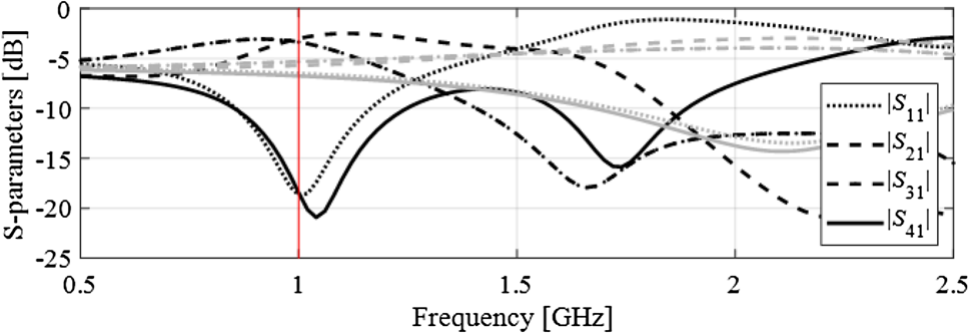
Branch-line coupler: scattering parameters at the initial (gray) and the final design (black) obtained using the proposed design specification management methodology. Target operating frequency marked using the vertical line.

**Figure 7:**
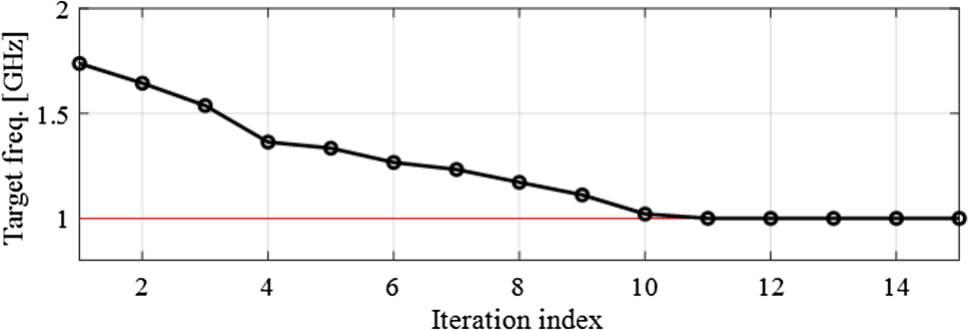
Branch-line coupler: evolution of the target frequency versus iteration index of the optimization algorithm. Original target frequency marked using a horizontal line.

**Figure 8:**
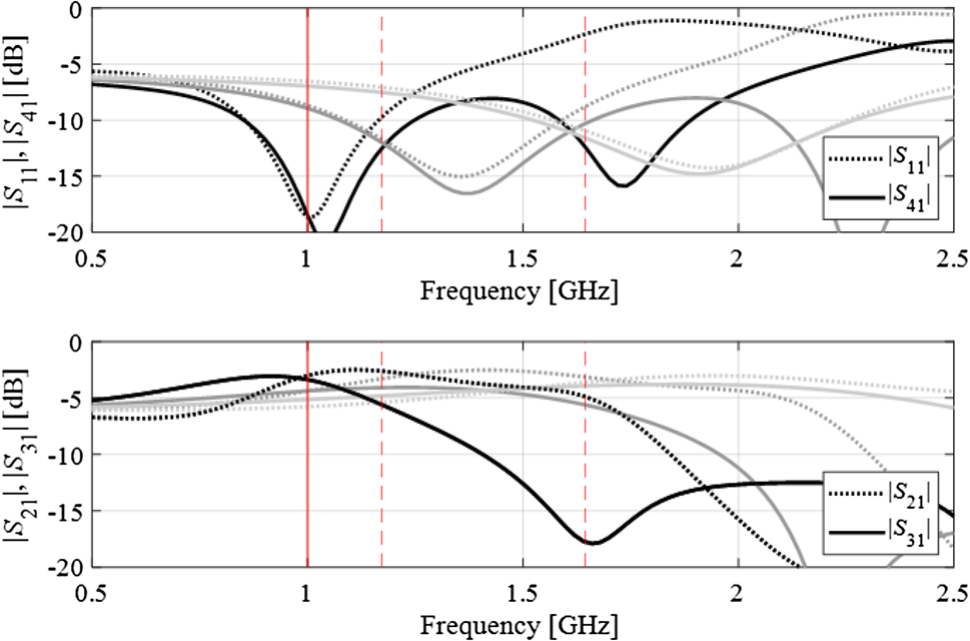
Branch-line coupler: scattering parameters at three intermediate designs, marked using the light-gray, dark-gray, and black colors (optimum), along with the corresponding target frequencies. For the sake of clarity, the matching/isolation characteristics are shown separate from the transmission characteristics.

The design problem is stated as follows. The circuit is to operate at the frequency *f*_0_ = 1 GHz, at which the matching |*S*_11_|, and isolation |*S*_41_| are to be simultaneously minimized. Also, the circuit has to ensure equal power split, i.e., |*S*_21_| =|*S*_31_| at *f*_0_. The objective function is formulated as in (2). Figure 6 shows the initial design selected for this problem (gray lines), as well as the final design obtained using the proposed approach (black lines). It can be observed that the operating frequency of the BLC at the initial design is at about 2.2 GHz, which makes it essentially impossible to re-design the structure to 1 GHz using local means. As a matter of fact, the conventional trust-region routine fails to do so. On the other hand, the proposed approach works well, and produces the design x^*^ = [1.00 0.81 6.91 11.94 0.75 0.99 0.89 0.65 4.05 0.53]^*T*^, also illustrated in Fig. 6. The evolution of the target operating frequency has been shown in Fig. 7. Initially, the specifications have to be severely adjusted in order to ensure a success of local search, then gradually converge to 1 GHz after about ten iterations of the optimization process. Some of the intermediate designs produced in the course of the optimization run have been illustrated in Fig. 8.

### Example 2: dual-band branch-line coupler

**Figure 9:**
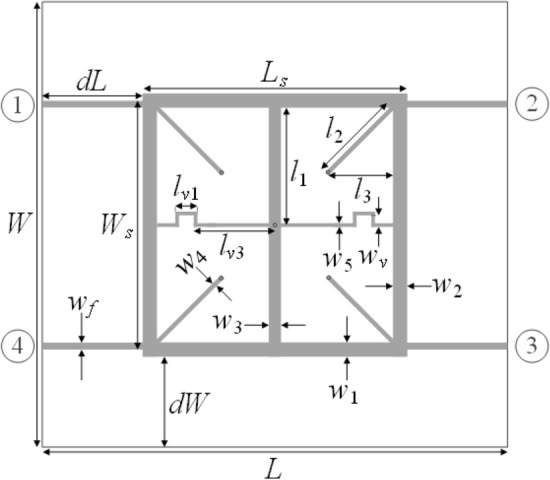
Dual-band branch-line coupler^56^; circuit topology; port marked with numbers in circles.

The second example is a dual-band branch line coupler^56^ shown in Fig. 9, implemented on the RO4003 substrate (*ε*_*r*_ = 3.5, *h* = 0.51 mm, tan*δ* = 0.0027). There circuit geometry is described by nine independent parameters ***x*** = [*L*_*s*_ *W*_*s*_ *l*_*3r*_ *w*_1_ *w*_2_ *w*_3_ *w*_4_ *w*_5_ *w*_*v*_]^*T*^ (all dimensions in mm, except l_3r_, which is a relative parameter). Furthermore, we have the following relationships: *d*_*L*_ = *d*_*W*_ = 10 mm, *L* = 2*d*_*L*_ + *L*_*s*_, *W* = 2*d*_*W*_ + 2*w*_1_ + (*W*_*s*_ − 2*w*_*f*_), *l*_1_ = *W*_*s*_/2, *l*_2_ = *l*_3_2^1/2^, *l*_3_ = *l*_3*r*_((*L*_*s*_ − *w*_3_)/2 − *w*_4_/2^1/2^), *l*_*v*1_ = *l*_3_/3, and *l*_*v*3_ = *L*_*s*_/2 − *w*_3_/2 − *l*_3_ + *l*_*v*1_; *w*_*f*_ = 1.15 mm is fixed to ensure 50-Ω line impedance. The computational model is implemented in CST Microwave Studio and evaluated using its time domain solver (~ 150,000 mesh cells, simulation time about 2 min).

For this example, the design objective is to optimize the geometry parameters so that the circuit operates at the frequencies *f*_1_ = 1.2 GHz and *f*_2_ = 2.7 GHz, with the input matching |*S*_11_|, and isolation |*S*_41_| simultaneously minimized at both *f*_1_ and *f*_2_. Furthermore, the circuit has to ensure equal power split, i.e., |*S*_21_| =|*S*_31_| at *f*_1_ and *f*_2_. The objective function for this problem is based on (2).

**Figure 10:**
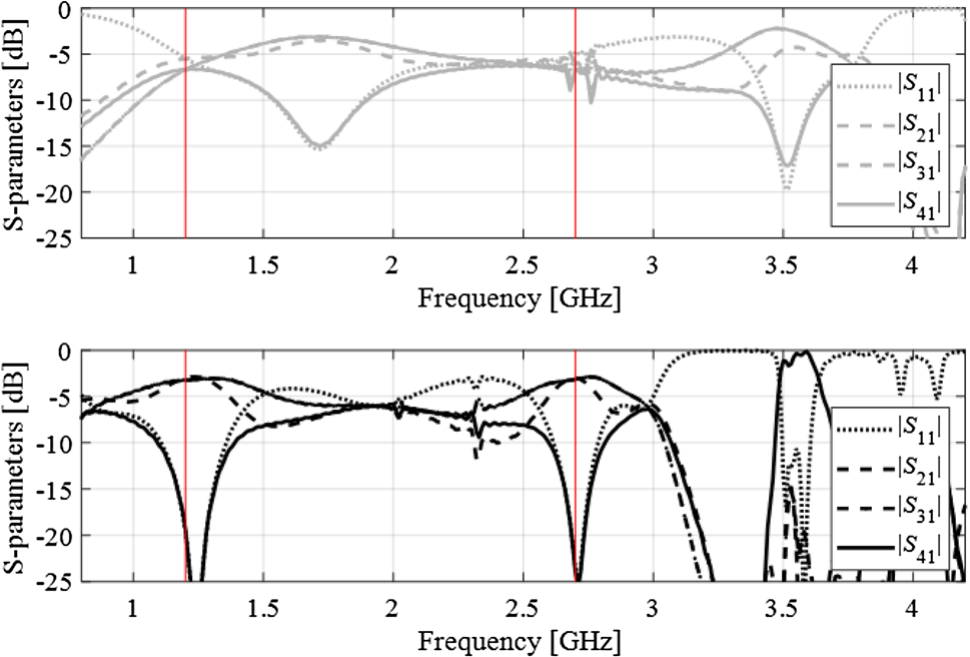
Dual-band branch-line coupler: scattering parameters at the initial (top) and the final design (bottom) obtained using the proposed design specification management methodology. Target operating frequency marked using the vertical lines.

**Figure 11:**
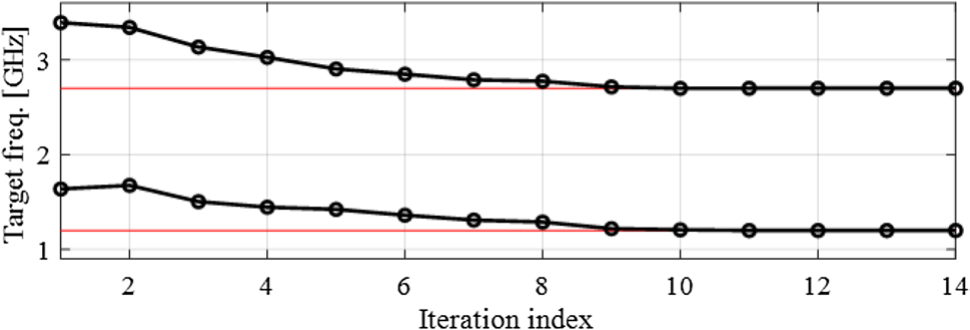
Dual-band branch-line coupler: evolution of the target operating frequencies versus iteration index of the optimization algorithm. Original target frequencies marked using a horizontal lines.

**Figure 12:**
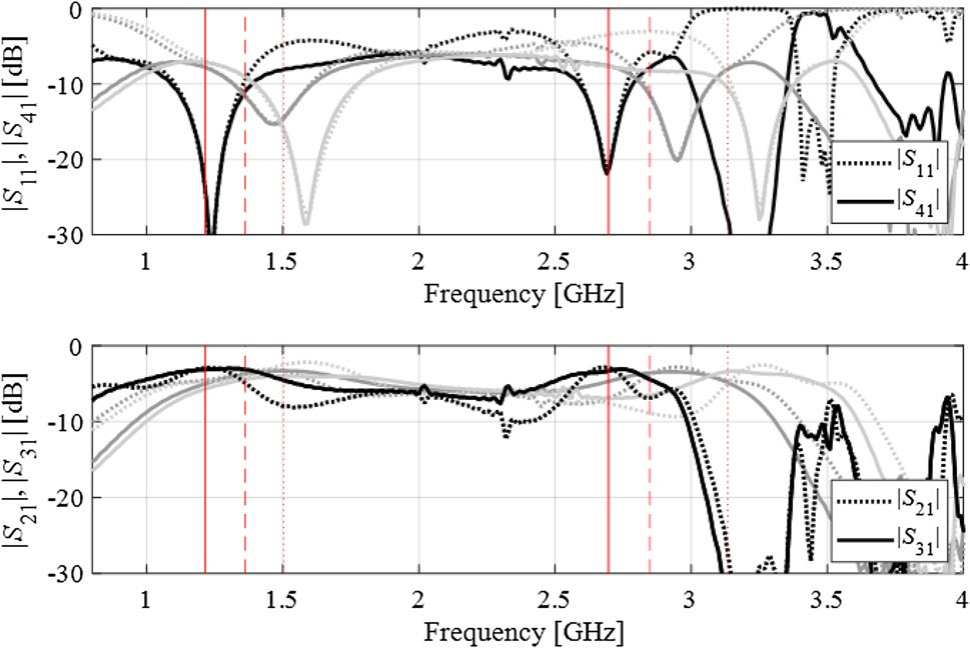
Dual-band branch-line coupler: scattering parameters at the three intermediate designs, marked using the light-gray, dark-gray, and black colors (optimum), along with the corresponding target frequencies. For the sake of clarity, the matching/isolation characteristics are shown separate from the transmission characteristics.

The initial design is shown in Fig. 10 using the gray lines. The operating frequencies at ***x***^(0)^ (around 1.7 GHz and 3.5 GHz, respectively) are severely misaligned with the target ones, and conventional local optimization fails to yield satisfactory results. The proposed approach renders the design x^*^ = [41.1 8.19 0.95 2.26 1.68 0.96 0.34 1.18 1.14]^*T*^ (responses marked black in Fig. 10) that is of high quality with respect to the assumed goals. The evolution of the target operating frequency has been shown in Fig. 11. Similarly as in the first test case, the specifications are considerably relocated and eventually converge to the original values after ten iterations of the algorithm. Figure 12 shows the selected intermediate designs along with the respective target operating frequencies.

### Example 3: dual-band power divider

**Figure 13:**
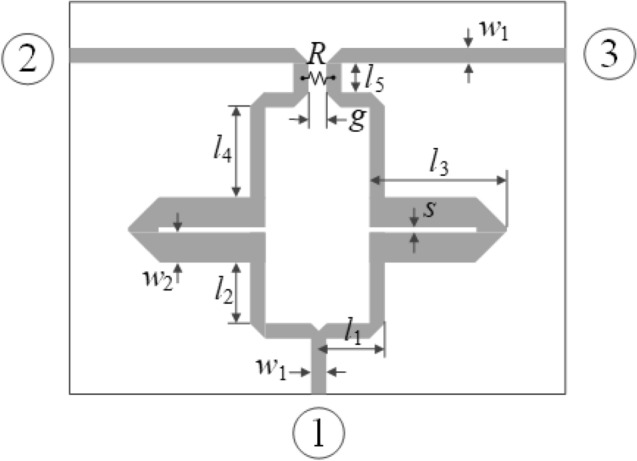
Dual-band equal split power divider^57^; circuit topology; port marked with numbers in circles. Lumped resistor denoted as *R*.

Our last example is a dual-band equal split power divider based on coupled lines^57^, shown in Fig. 13. The circuit is implemented on AD250 dielectric substrate (ε_*r*_ = 2.5, *h* = 0.81 mm, tanδ = 0.0018). There are seven adjustable parameters ***x*** = [*l*_1_ *l*_2_ *l*_3_ *l*_4_ *l*_5_ *s w*_2_]^*T*^ (all dimensions in mm); *w*_1_ = 2.2 is fixed to ensure 50-Ω line impedance; *g* = 1 mm is also fixed. The computational model is implemented in CST Microwave Studio and evaluated using its time domain solver (~ 200,000 mesh cells, simulation time approx. 2 min).

**Figure 14:**
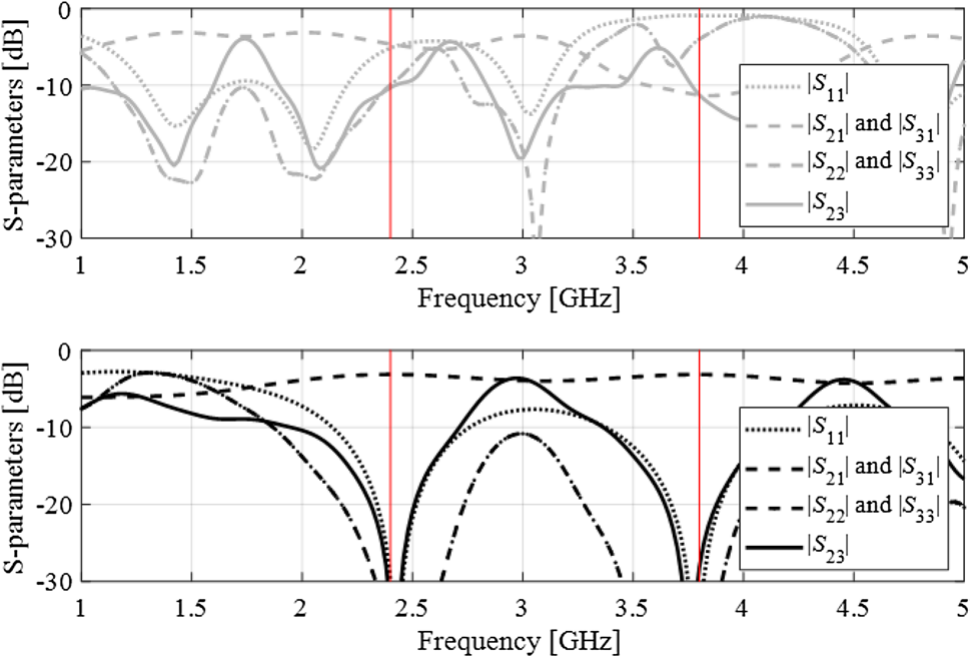
Dual-band power divider: scattering parameters at the initial (top) and the final design (bottom) obtained using the proposed design specification management methodology. Target operating frequencies marked using the vertical lines.

The design objective is to optimize the geometry parameters so that the circuit operates at the frequencies *f*_1_ = 2.4 GHz and *f*_2_ = 3.8 GHz, with the input matching |*S*_11_|, output matching |*S*_22_|, |*S*_33_|, as well as isolation |*S*_23_| simultaneously minimized at both *f*_1_ and *f*_2_. The equal power split condition is not directly handled in the optimization process because it is implied by the structure symmetry. The initial design is shown in Fig. 14 using the gray lines). The operating frequencies at ***x***^(0)^ (around 1.4 GHz and 2.0 GHz, respectively) are away from the target ones. Similarly as for the two previous examples, conventional local optimization fails to produce satisfactory results. The methodology proposed in this work yields the design x^*^ = [26.86 2.18 21.92 2.00 3.82 0.50 4.56]^*T*^ (marked black in Fig. 14), which meets the performance requirements imposed on the structure.

**Figure 15:**
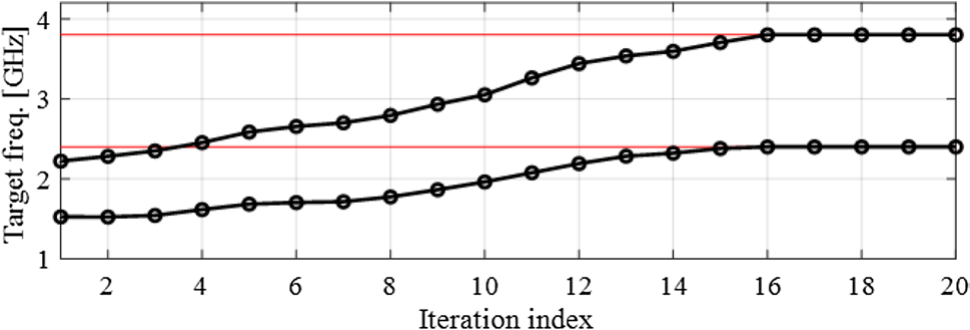
Dual-band power divider: evolution of the target operating frequencies versus iteration index of the optimization algorithm. Original target frequencies marked using a horizontal lines.

**Figure 16:**
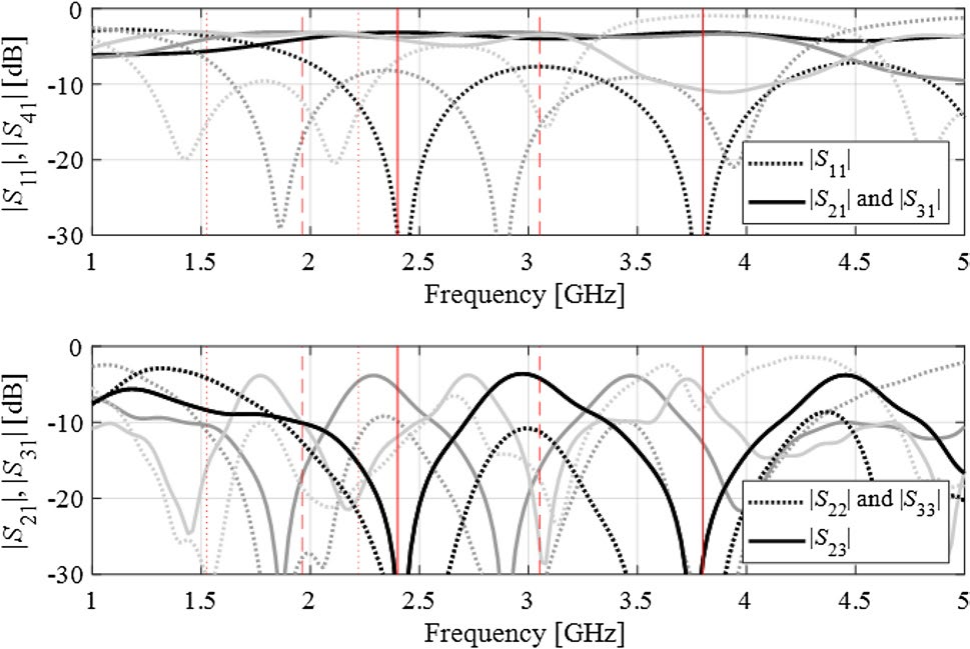
Dual-band power divider: scattering parameters at the three intermediate designs, marked using the light-gray, dark-gray, and black colors (optimum), along with the corresponding target frequencies. For clarity, the input matching and transmission responses, as well as the output matching and isolation responses are shown in separate panels.

The evolution of the target operating frequencies has been shown in Fig. 15. It is consistent with what was observed in previous sections: following the initial (and significant) adjustment, the target frequencies converge to their original values towards the end of the optimization process. Figure 16 shows the selected intermediate designs along with the respective target operating frequencies.

## Conclusion

In this work, a novel design specification adjustment procedure through intelligent decision-making has been introduced to improve the reliability of EM-driven optimization of miniaturized microwave components. The procedure is intended to be used with local iterative search procedures (primarily gradient-based routines of a descent type), and to alleviate the difficulties related to the lack of reasonable initial designs. The proposed approach allows for automated and adaptive modification of the design objectives in terms of the target operating frequencies or bandwidths, so that the relocated targets are attainable at any given stage of the optimization process from the currently available design. Essentially, it is a knowledge-based methodology that employs several indicators related to the convergence status of the optimization process, frequency spread of the system characteristics, and quantified misalignment between the actual and required operating conditions. Rigorous criteria have been defined and implemented to activate the design specification adjustment, and to decide about the amount thereof. Our technique—upon coupling with the trust-region gradient-based algorithm—has been comprehensively validated using three microstrip circuits, a single-band and a dual-band branch-line couplers, and a dual-band power divider. In all considered cases, the optimization process was demonstrably convergent to satisfactory designs despite of poor starting points, at which the operating frequencies of the respective structures have been severely misaligned with the target ones. Based on these results, it can be concluded that the presented methodology significantly improves reliability of the optimization procedure.

The principal advantage of the proposed intelligent design specification management is a reduction of the optimization process sensitivity to the quality of the initial design. Some of the practically important consequences include a possibility of re-designing (dimension scaling) of microwave components within broad ranges of operating conditions using local procedures, as well as limiting the need for global search algorithms (or various advanced approaches such as surrogate-assisted or machine learning frameworks) when handling more challenging parameter tuning tasks. Furthermore, the presented technique allows for replacing heuristic and interactive (experience-driven) methods of design specifications adjustment, effectively leading to a considerable shortening of design closure cycles under challenging scenarios.
